# *RNF213* gene mutation in circulating tumor DNA detected by targeted next‐generation sequencing in the assisted discrimination of early‐stage lung cancer from pulmonary nodules

**DOI:** 10.1111/1759-7714.13741

**Published:** 2020-11-16

**Authors:** Ning Jiang, Jie Zhou, Wenhao Zhang, Peichao Li, Yu Liu, Hubo Shi, Chengke Zhang, Yunshan Wang, Chengjun Zhou, Chuanliang Peng, Weiquan Zhang, Yingtao Hao, Qifeng Sun, Yuliang Li, Xiaogang Zhao

**Affiliations:** ^1^ Department of Thoracic Surgery The Second Hospital of Shandong University Jinan China; ^2^ Key Laboratory of Chest Cancer Shandong University Jinan China; ^3^ Cheeloo College of Medicine Shandong University Jinan China; ^4^ Department of Thoracic Surgery The 960th Hospital of People's Liberation Army of China Jinan China; ^5^ Department of Thoracic Surgery Shandong Provincial Chest Hospital Jinan China; ^6^ Department of Clinical Laboratory The Second Hospital of Shandong University Jinan China; ^7^ Pathology Department The Second Hospital of Shandong University Jinan China; ^8^ Department of Thoracic Surgery Shandong Provincial Hospital Jinan China; ^9^ Department of Interventional Medicine The Second Hospital of Shandong University Jinan China; ^10^ Interventional Oncology Institute, Shandong University Jinan China

**Keywords:** Circulating tumor DNA (ctDNA), early diagnosis, lung cancer, *RNF213* gene, targeted next generation sequencing (NGS)

## Abstract

**Background:**

To distinguish early‐stage lung cancer from benign disease in pulmonary nodules, especially lesions with ground‐glass opacity (GGO), we assessed gene mutations of ctDNA in peripheral blood using targeted next‐generation sequencing (NGS).

**Methods:**

Single pulmonary nodule patients without mediastinal lymph nodes and symptoms that were hard to diagnose by chest CT and lung cancer biomarker measurement in multiple medical centers were enrolled into the study. All patients accepted minimally invasive surgery but refused preoperative biopsy. Gene mutations in preoperative blood samples were detected by targeted NGS. Mutations with significant differences between lung tumors and benign lesions, as grouped by postoperative pathology, were screened. Protein expression was determined by immunohistochemistry. Highly expressed genes were selected as biomarkers to verify the mutations in peripheral blood.

**Results:**

In the training set, the *RNF213*, *KMT2D*, *CSMD3* and *LRP1B* genes were mutated more frequently in early‐stage lung cancer (27 cases) than in benign nodules (15 cases) (*P* < 0.05). High expression of the *RNF213* gene in lung cancers and low expression in benign diseases were seen by immunohistochemistry. The *RNF213* gene was mutated in 25% of lung cancer samples in the validation set of 28 samples and showed high specificity (100%). In GGO patients, *RNF213* was mutated more frequently in early‐stage lung cancer compared to benign diseases (*P* < 0.05).

**Conclusions:**

*RNF213* gene mutations were observed more frequently in early‐stage lung cancer, but not in benign nodules. Mutation of the *RNF213* gene in peripheral blood may be a high specificity biomarker for the assisted early diagnosis of lung cancer in pulmonary nodules.

**Key points:**

Significant findings of the study: In peripheral venous blood and tumor tissue, *RNF213* gene mutated more frequently in lung cancer than benign pulmonary nodules.

What this study adds: Detection mutation of the *RNF213* gene in peripheral blood may be a high specificity method for the assisted early diagnosis of lung cancer in pulmonary nodules.

## Graphical abstract

We found a novel *RNF213* gene with high specificity in peripheral blood ctDNA in assisted diagnosis of lung cancer. It may be helpful about assisted diagnosis and treatment of lung cancer.

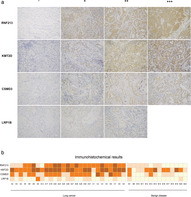

## Introduction

Lung cancer remains a life‐threatening malignancy with the highest morbidity and mortality of any cancer across the world.^1^ The five‐year survival of lung cancer patients is still low,^2^, ^3^ despite the use of molecular diagnosis and targeted therapy. Early diagnosis and treatment are effective ways to improve the survival of lung cancer patients. Using low‐dose computed tomography (LDCT) in screening can reduce lung cancer‐related mortality, and smaller pulmonary nodules may be found at an early stage. However, the diagnosis may be difficult in some cases with atypical CT imaging, and traditional biomarkers such as carcinoembryonic antigen (CEA), neuron‐specific enolase (NSE) and cytokeratin 19 (CYFRA‐211) might not be positive early on. Aspiration biopsy or surgery may be needed in most patients to confirm whether the nodules are malignant or benign.

The ideal diagnostic method should be simple, less traumatic, and easy to obtain and have a high positive rate. Circulating cell‐free DNA (cfDNA) is a fragment of DNA released through cell apoptosis that widely exists in blood, cerebrospinal fluid, urine and saliva.^4^, ^5^ As cfDNA can also be released by tumor cells through apoptosis and necrosis,^6^, ^7^ this DNA is also called circulating tumor DNA (ctDNA). Liquid biopsy of the blood for ctDNA detection is important in the diagnosis, monitoring and prognosis of the tumor.^8^

The patient's ctDNA is meaningful to better understand the disease. ctDNA reflects the somatic genetic features of the primary tumor.^9^ It can be detected in the peripheral blood of patients with advanced cancers and can be used for monitoring therapeutic effects.^10^, ^11^ The content of plasma ctDNA accounts for 0.01% of cfDNA.^12^ Studies^13^ have indicated that the concentration of ctDNA in the plasma increases with stage, probably because of the increasing tumor burden. Very low levels of detectable ctDNA in plasma and unknown mutations have limited the potential application in the diagnosis of early‐stage lung cancer.

With the increasing sensitivity of next‐generation sequencing (NGS), low concentrations of ctDNA in blood can be detected. At present, ctDNA of advanced‐stage lung cancer has been studied in blood to monitor therapeutic effects. Few studies have studied early‐stage lung cancer by detecting tumor DNA in tissue or identifying mutations in ctDNA in lung cancer patients, and this has been done in a limited number of genes.^14^ Some lung cancer‐related genes, such as *EGFR*, *ALK*, and *KRAS*, are usually used for targeted NGS in early‐stage lung cancer.^15^, ^16^ Only a few genes from the panel are used for targeted NGS. In addition, healthy or benign‐nodule individuals need to be used as the control group. To date, no study has addressed whether ctDNA can be detected in benign pulmonary nodules or whether there are differences in ctDNA in undiagnosed pulmonary nodules, including early‐stage lung cancer and benign disease.

Here, we study ctDNA through targeted NGS in pulmonary nodules that could not be clearly diagnosed by chest CT. A panel of 560 tumor‐related hot‐spot genes was used to evaluate the targeted sequencing of plasma ctDNA in malignant and benign pulmonary nodules. We aimed to identify discrepant ctDNAs in the two groups to guide early diagnosis in lung cancer.

## Methods

### Patients

Patients with single pulmonary nodules were diagnosed in 2017–2018 and enrolled in the study at the Second Hospital of Shandong University, Shandong Provincial Chest Hospital and the 960th Hospital of the People's Liberation Army of China. Lung cancer or benign disease could not be confirmed by chest CT. The largest diameter of the lesion was less than 4 cm, and there was no involvement of mediastinal lymph nodes on CT imaging. The cTNM stage was less than cT2aN0M0 (stage I, TNM staging manual eighth edition) if the lesion was considered to be lung cancer. In terms of preoperative routine examination, there were no metastatic lesions and no patients with any other oncological history. Lung cancer‐related biomarkers, such as CEA, NSE and CYFRA‐211, could not assist with a definitive diagnosis in patients. All patients refused biopsy or it was difficult to obtain tissues for histological diagnosis. All patients accepted minimally invasive thoracoscopic surgery.

### Study design

A training set was established. In accordance with the uniform diagnostic criteria, inclusion criteria and exclusion criteria, 42 of 58 registered patients met the standard and passed the blood sample test. Plasma and leukocytes in the blood sample were separated, and DNA in leukocytes was used as a self‐control to exclude germline mutations. Qualified paired plasma and leukocyte samples were sequenced by targeted NGS with a panel of 560 tumor‐related hot‐spot mutant genes. Mutated ctDNA was analyzed in lung cancer and benign disease according to histopathological results. We selected significantly different ctDNAs in the lung cancer group compared to the benign disease control group. Immunohistochemical staining was performed in formalin‐fixed, paraffin‐embedded (FFPE) tissue samples of these patients to analyze the expression of the selected ctDNAs. Finally, high expression of the selected ctDNAs in lung cancer was confirmed. A validation set including unknown pathological pulmonary nodules was established and sequenced by the same panel NGS to test the selected ctDNA mutations.

### Blood sample preparation

Peripheral blood (10 mL)was sampled one to three days before the operation. Blood samples in EDTA tubes were centrifuged for 10 minutes at 1600 *g* at 4°C, and white blood cells were collected and stored. The supernatants from these samples were further centrifuged at 16000 *g* for 10 minutes at 4°C, and plasma was collected and stored at −80°C until use. White blood cell DNA was isolated using the DNA Isolation Kit for Mammalian Blood (Roche), and cfDNA was isolated using the QIAamp Circulating Nucleic Acid Kit (QIAGEN) according to the manufacturer's protocol. A total of 100–300 ng cfDNA was acquired from 1 mL plasma.

### Genomic DNA preparation and targeted sequencing

The quality of genomic DNA in terms of degradation and contamination was monitored on a 1% agarose gel, and the concentration was measured using a Qubit DNA Assay Kit in Qubit 2.0 Fluorometer (Life Technologies, Carlsbad, CA, USA).

We designed probes on the website of Agilent for particular genes according to the design instructions to cover the target gene regions. Briefly, 180–280 bp fragments were produced from fragmentation carried out by a hydrodynamic shearing system (Covaris, Woburn, Massachusetts, USA). Extracted DNA was then amplified by ligation‐mediated PCR (LM‐PCR), purified, and hybridized to the probe for enrichment. Nonhybridized fragments were subsequently washed out. Both captured and noncaptured LM‐PCR products were subjected to real‐time PCR to estimate the magnitude of enrichment. High‐throughput sequencing was carried out at the average 1000× sequence depth when each captured library was loaded on an Illumina HiSeq 4000 platform (Illumina, San Diego, California, USA). Each captured library was sequenced independently to ensure that each sample met the desired average fold coverage.

### Sequence data quality control

The original fluorescence image files obtained from the HiSeq platform were transformed to short reads (raw data) by base calling and recorded in FASTQ format, which contained sequence information and corresponding sequencing quality information. Clean reads were acquired by excluding reads containing adapter contamination and low‐quality/unrecognizable nucleotides. Downstream bioinformatic analyses were based on these clean data. At the same time, the total read number, sequencing error rate, percentage of reads with average quality >20, percentage of reads with average quality >30, and GC content distribution were calculated.

### Read mapping and somatic genetic alteration detection

Valid sequencing data were mapped to the reference human genome (UCSC hg19) by Burrows‐Wheeler Aligner (BWA) software to obtain the original mapping results, stored in BAM format.^17^ Then, SAM tools^18^ and Picard (http://broadinstitute.github.io/picard/) were used to sort BAM files and perform duplicate marking, local realignment, and base quality recalibration to generate a final BAM file for computing the sequence coverage and depth.

MuTect and Strelka software^19^, ^20^ were used for calling somatic single‐nucleotide variations (SNVs) and small insertions and deletions (InDels) from paired tumor/normal samples. In addition to default filters, polymorphisms of somatic SNVs and InDels referenced in the 1000 Genomes Project^21^ or Exome Aggregation Consortium (ExAC)^22^ with a minor allele frequency over 1% were removed. Subsequently, VCF (variant call format) data were annotated by ANNOVAR software.^23^

### Immunohistochemical (IHC) analysis

Immunohistochemical (IHC) analysis was performed on 5 μm thick sections derived from formalin‐fixed and paraffin‐embedded lung cancer and benign disease tissue samples. In brief, all the slides were dewaxed with xylene and a graded ethanol series, antigen was repaired in citrate buffer (Beyotime Institute of Biotechnology, Haimen, China), nonspecific binding was blocked with 1.5% goat serum (Beyotime Institute of Biotechnology, Shanghai, China), and the slides were then incubated with the primary antibodies at 4°C overnight. Primary antibodies against KMT2D (Cat No. 27266‐1‐AP) and RNF213 (Cat No. 21028‐1‐AP) were obtained from Proteintech Group (Wuhan, Hubei, China). Primary antibodies against LRP‐1B (Cat No. NBP2‐49582) and CSMD3 (Cat No. NBP1‐86371) were purchased from Novus Biologicals (Centennial, CO, USA). The slides were then washed and stained with the secondary antibody (goat anti‐rabbit IgG H&L (HRP), cat. No. ab205718; Abcam) and DAB, counterstained with hematoxylin, dehydrated and mounted. The results were evaluated independently by two independent pathologists.

Membranous or cytoplasmic staining for KMT2D, RNF213, LRP‐1B and CSMD3 was defined as positive. The staining intensity and extent of the staining area were graded according to a semiquantitative scoring system. Staining intensity was characterized as follows: 0, none; 1, weak; 2, intermediate; and 3, strong. The extent of staining was defined as 0, none; 1, <1/100; 2, 1/100 to 1/10; 3, 1/10 to 1/3; 4, 1/3 to 2/3; and 5, >2/3 of cells expressing the respective lesions.^24^ The final total score was achieved by adding the scores of intensity and extent and ranged from 0 to 8. Scores of 0 were defined as “−,” scores of 2–4 were defined as “+,” scores of 5–6 were defined as “++” and scores of 7–8 were defined as “+++.” Low expression was defined as scores of 0–4 (“−”and “+”), and high expression was defined as scores of 5–8 (“++”and “+++”).

### Statistical analysis

BWA, Samblaster and Sambamba software were used to compare the sequenced data with the reference genome. MuTect software was used to search for somatic single‐nucleotide variation (SNV) mutations. Strelka software was used to search for somatic insertions‐deletions (InDels). ANNOVAR software was used to annotate the structure and function of the detected variations. Lung cancer and benign disease were divided into two groups. Data are presented as mean and standard error of mean (mean ± SEM). Categorical data were analyzed using Pearson's chi‐square test or Fisher's exact test, and continuous data were analyzed by Student's *t*‐test or one‐way ANOVA. Analyses were performed using SPSS Statistics version 23 (IBM Corp). A two‐tailed *P*‐value <0.05 was considered to be statistically significant.

## Results

### Patient general characteristics

In the training set, a total of 42 pairs of qualified samples were sequenced after rejecting contaminated genome and unqualified samples, including samples of patients diagnosed with stage III lung cancer according to postoperative pathology. The patients' general characteristics are shown in Table 1.

**Table 1:** Patients' characteristics in training set

	Lung cancer No. (%)	Benign disease No. (%)		*P*‐value
Number	27 (64.3%)	15 (35.7%)		
Gender				1.000
Male	18 (42.9%)	10 (23.8%)		
Female	9 (21.4%)	5 (11.9%)		
Age	44–73	33–72		0.001
Smoking				0.611
Smoker	13 (31.0%)	6 (14.3%)		
Nonsmoker	14 (33.3%)	9 (21.4%)		
Tumor size				1.000
≤3 cm	22 (52.4%)	12 (28.6%)		
3 cm<T†<4 cm	5 (11.9%)	3 (7.1%)		
Pathology				
Ad	18 (42.9%)	Tuberculosis	3 (7.1%)	
SC	7 (16.7%)	Inflammation	9 (21.4%)	
Others	2 (4.8%)	Hamartoma	2 (4.8%)	
		SH	1 (2.4%)	
Stage				
IA	8 (19.0%)			
IB	14 (33.3%)			
IIA	0 (0%)			
IIB	5 (11.9%)			

† Ad, adenocarcinoma；Others, large cell carcinoma, sarcomatoid carcinoma; SC, squamous carcinoma；SH, sclerosing hemangioma; T, tumor.

There were 27 lung cancer and 15 benign disease patients in the training set, including 18 male and nine female patients in the lung cancer group and 10 male and five female patients in the benign disease group. The average ages of the two groups were 63.1 ± 1.7 and 52.3 ± 2.8, respectively (*P* < 0.05). The numbers of stage I and stage II lung cancer patients were 22 (52.4%) and five (11.9%), respectively. Grouping by tumor size, there were 22 lung cancer patients (52.4%) and 12 benign disease patients (28.6%) in the group with a diameter less than 3 cm, while those with a diameter of 3 to 4 cm were five (11.9%) and three (7.1%). There was no significant difference (*P* > 0.05) in the general characteristics between the two groups, including sex, smoking history and tumor size.

The pathological type of lung cancer included adenocarcinoma (18, 42.9%), squamous carcinoma (7, 16.7%) and other types (2, 4.8%), which were large cell and sarcomatoid carcinomas. The histology of most lung cancer patients was adenocarcinoma. In benign lung disease, the postoperative pathological types were tuberculosis (3, 7.1%), inflammatory pseudotumor (9, 21.4%), hamartoma (2, 4.8%) and sclerosing hemangioma (1, 2.4%). The numbers of stage IA, IB, IIA and IIB lung cancer were eight (19.0%), 14 (33.3%), 0 (0%) and five (11.9%), respectively. There was no significant difference in any general data (*P* > 0.05) except age.

### Biomarker detection

Blood biomarkers, including CEA, NSE and CYFRA‐211, were detected before surgery. In the training set, the average values of these biomarkers were 2.45 ± 0.30, 18.44 ± 2.72, and 3.14 ± 1.10 ng/mL in lung cancer and 1.89 ± 0.22, 18.73 ± 1.80, and 1.73 ± 0.21 ng/mL in benign disease, respectively, and there were no significant differences (*P* > 0.05 for each comparison) in the two groups. In the validation set, the average values of these biomarkers were 2.75 ± 0.35, 13.93 ± 1.06, 2.19 ± 0.22 ng/mL in lung cancer and 1.72 ± 0.36, 15.46 ± 1.63, 2.18 ± 0.29 ng/mL in the benign disease groups, and there were no significant differences (*P* > 0.05) between the two groups.

Positive staining of the biomarkers was judged according to the range of clinical reference values. In the training set, there were 3/27 and 0/15 CEA‐positive, 6/27 and 6/15 NSE‐positive, and 2/27 and 0/15 CYFRA‐211‐positive patients, respectively, in the lung cancer and benign disease groups (*P* > 0.05 for all three biomarkers). In the validation set, there were 1/20 and 0/8 CEA‐positive, 4/20 and 1/8 NSE‐positive, and 3/20 and 1/8 CYFRA‐211‐positive patients, respectively, in the lung cancer and benign disease groups (*P* > 0.05 for all three biomarkers) (Table 2).

**Table 2:** Positive biomarkers of two groups in the training and validation sets

	Training set			Validation set		
Biomarker	LC	BD	*P*‐value	LC	BD	*P*‐value
CEA	3/27	0/15	0.541	1/20	0/8	1.000
NSE	6/27	6/15	0.222	4/20	1/8	1.000
CYFRA‐211	2/27	0/15	0.530	3/20	1/8	1.000

BD, benign disease; LC, lung cancer.

### Cell‐free DNA detected

Cell‐free DNA (cfDNA) was detected in all 42 samples in the training set. The concentrations of cfDNA in the lung cancer group and benign disease group were 0.2–3.04 ng/μL and 0.21–1.25 ng/μL, respectively, and the average concentrations were 0.53 ± 0.13 ng/μL and 0.54 ± 0.07 ng/μL, respectively. The differences were not significant between the two groups (*P* > 0.05).

### Somatic mutation analysis

**Figure 1:**
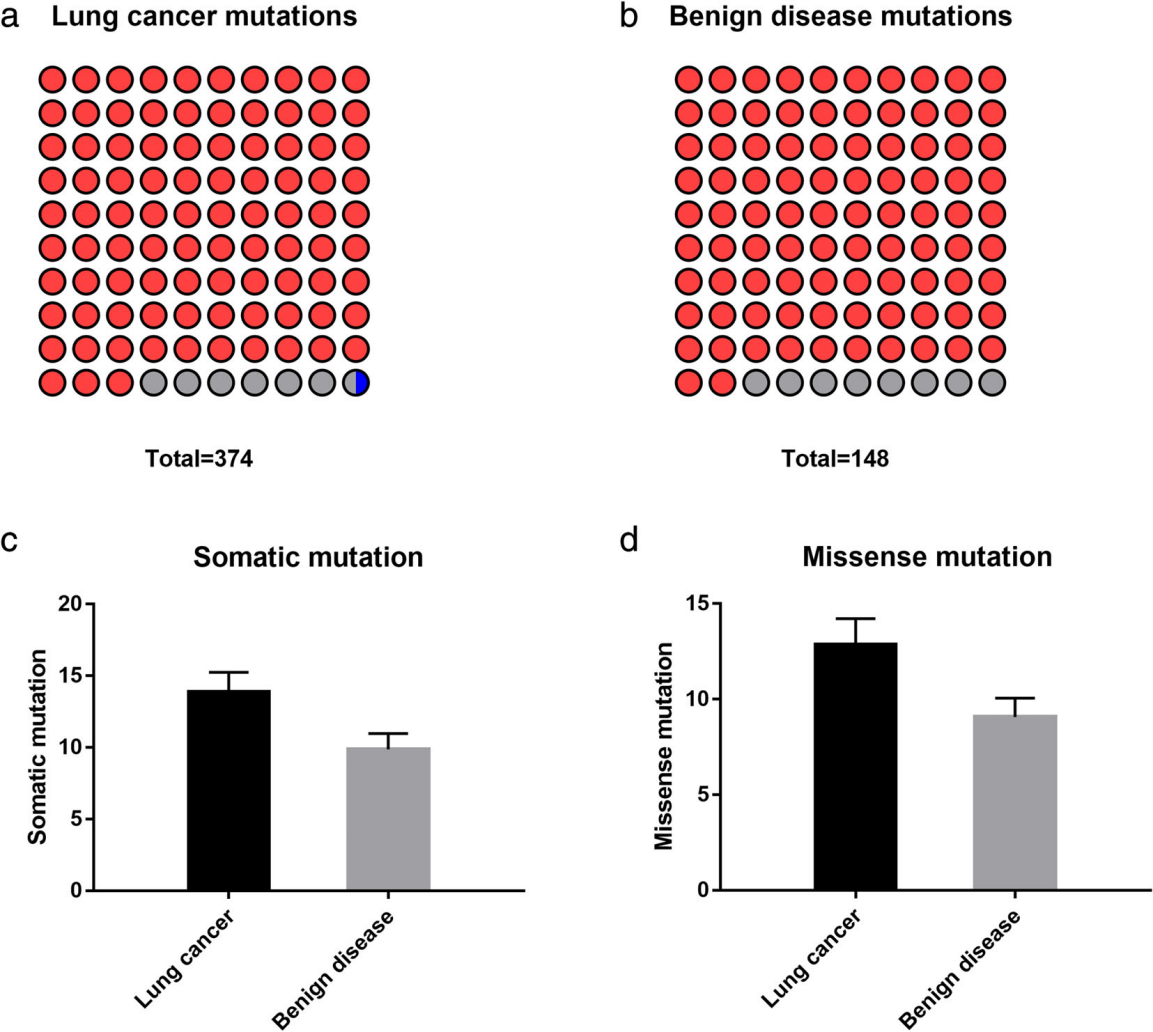
Mutations in the training set. (**a**) In the lung cancer group, 92.78% of mutations were missense mutations, 6.95% of mutations were nonsense mutations, and 0.27% of mutations were deletions. (**b**) In the benign lung disease group, 91.89% of mutations were missense mutations, and 8.11% of mutations were nonsense mutations. The number of somatic mutations (**c**) and missense mutations (**d**) in the two groups, and there were no significant differences between lung cancer and benign disease (*P* > 0.05). (**a**) (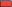) Missense mutation (92.78%), (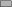) Nonsense mutation (6.95%), (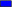) Deletion (0.27%); (**b**) (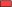) Missense mutation (91.89%), (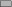) Nonsense mutation (8.11%)

The number of mutated genes by targeted sequencing was 246 in the lung cancer and benign disease groups in the training set. There was a total of 522 somatic mutations in the two groups, including 374 somatic mutations detected in the lung cancer group and 148 mutations in the benign disease group (Fig 1).

Most of the mutations were single‐nucleotide variations (SNVs). In the lung cancer group, there were 348 nonsynonymous mutations, including 347 missense mutations and only one deletion mutation (InDel), out of all 374 somatic mutations. In the benign disease group, there were 136 nonsynonymous mutations (missense mutations) in all 148 somatic mutations. The average numbers of somatic mutations were 13.85 ± 1.40 and 9.87 ± 1.10 in the two groups, respectively, and the missense mutations were 12.89 ± 1.35 and 9.07 ± 0.99, respectively. The differences were not significant between the two groups (*P* > 0.05) (Fig 1).

**Figure 2:**
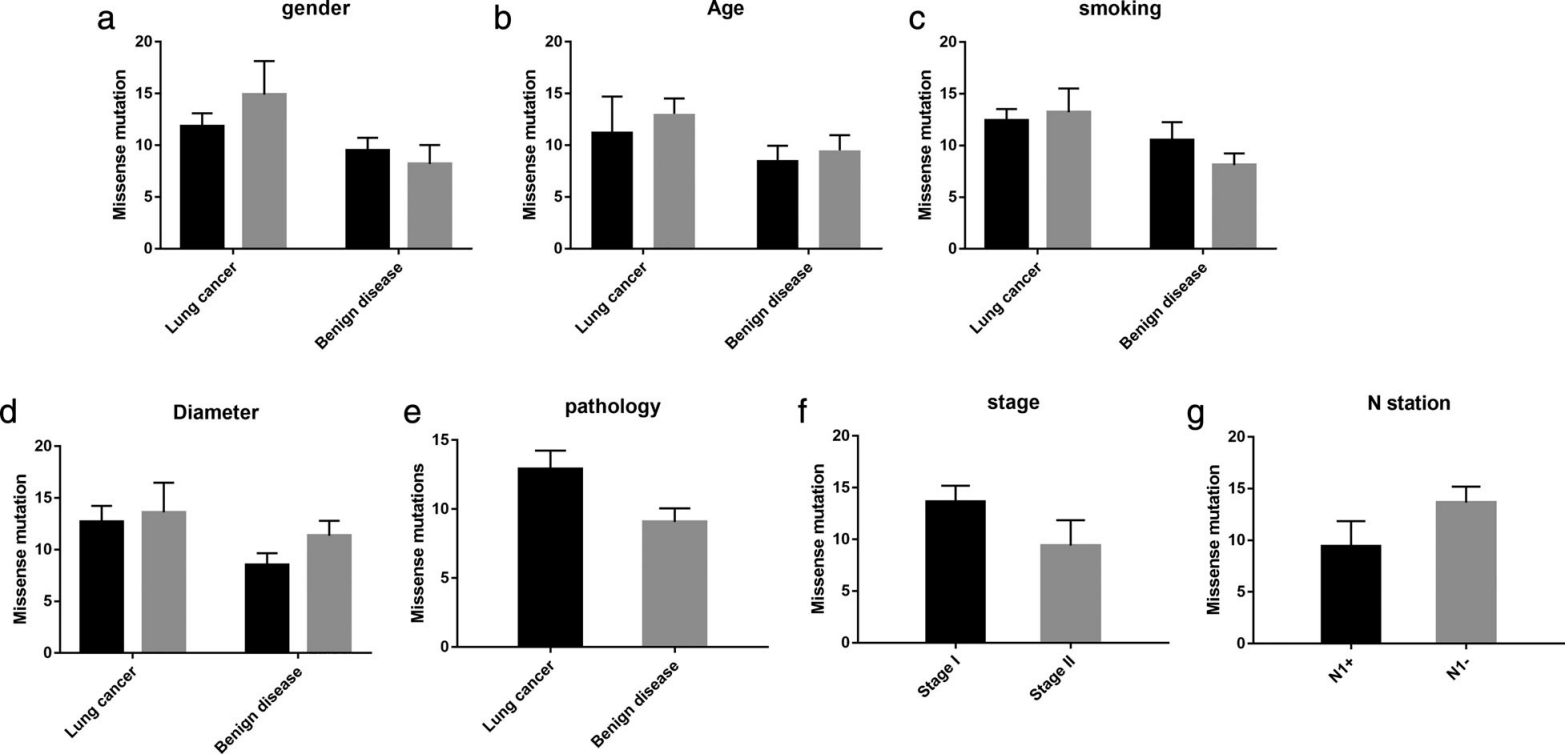
The number of missense mutations in the two groups. (**a**) In the lung cancer group, mutations in males and females were 11.83 ± 1.26 and 14.89 ± 3.25, respectively. In the benign lung disease group, mutations in males and females were 9.5 ± 1.22 and 8.20 ± 1.83, respectively. (**b**) In the lung cancer group, mutations in patients aged less than 50 years and more than 50 years were 11.33 ± 3.38 and 13.04 ± 1.49, respectively. In the benign diseases group, mutations in patients aged less than 50 years and more than 50 years were 8.57 ± 1.39 and 9.50 ± 1.47, respectively. (**c**) In the lung cancer group, there were 12.42 ± 1.11 mutations in smokers and 13.20 ± 2.33 mutations in nonsmokers. In the benign disease group, there were 10.50 ± 1.76 mutations in smokers and 8.11 ± 1.14 mutations in nonsmokers. (**d**) Mutations in the group with lung tumors less than 3 cm and 3–4 cm in diameter were 12.68 ± 1.57 and 13.60 ± 2.87, respectively. Mutations in patients with benign lesions less than 3 cm and 3–4 cm in diameter were 8.50 ± 1.15 and 11.33 ± 1.45, respectively. (**e**) Mutations in lung cancer and benign disease were 12.88 ± 1.35, 9.07 ± 0.99. (**f**) The number of missense mutations in stage I and stage II lung cancer was 13.64 ± 1.55 and 9.40 ± 2.46, respectively. (**g**) The number of missense mutations in N1 lymph node‐positive and ‐negative patients was 9.40 ± 2.46 and 13.64 ± 1.55, respectively. There were no statistically significant differences in any of the comparisons (*P* > 0.05). (**a**) (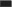) Male, (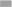) Female; (**b**) (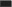) ≤50, (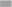) >50; (**c**) (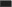) Smoking, (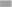) No smoking; (**d**) (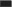) ≤3 cm, (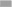) 3–4 cm

Analyzing the number of missense mutations against the general characteristics of the two groups, including the patients' sex, age, smoking history, tumor size, pathology, stage and N1 station lymphatic metastasis, there were no statistically significant correlations (*P* > 0.05) (Fig 2).

### ctDNA detected by targeted NGS

**Figure 3:**
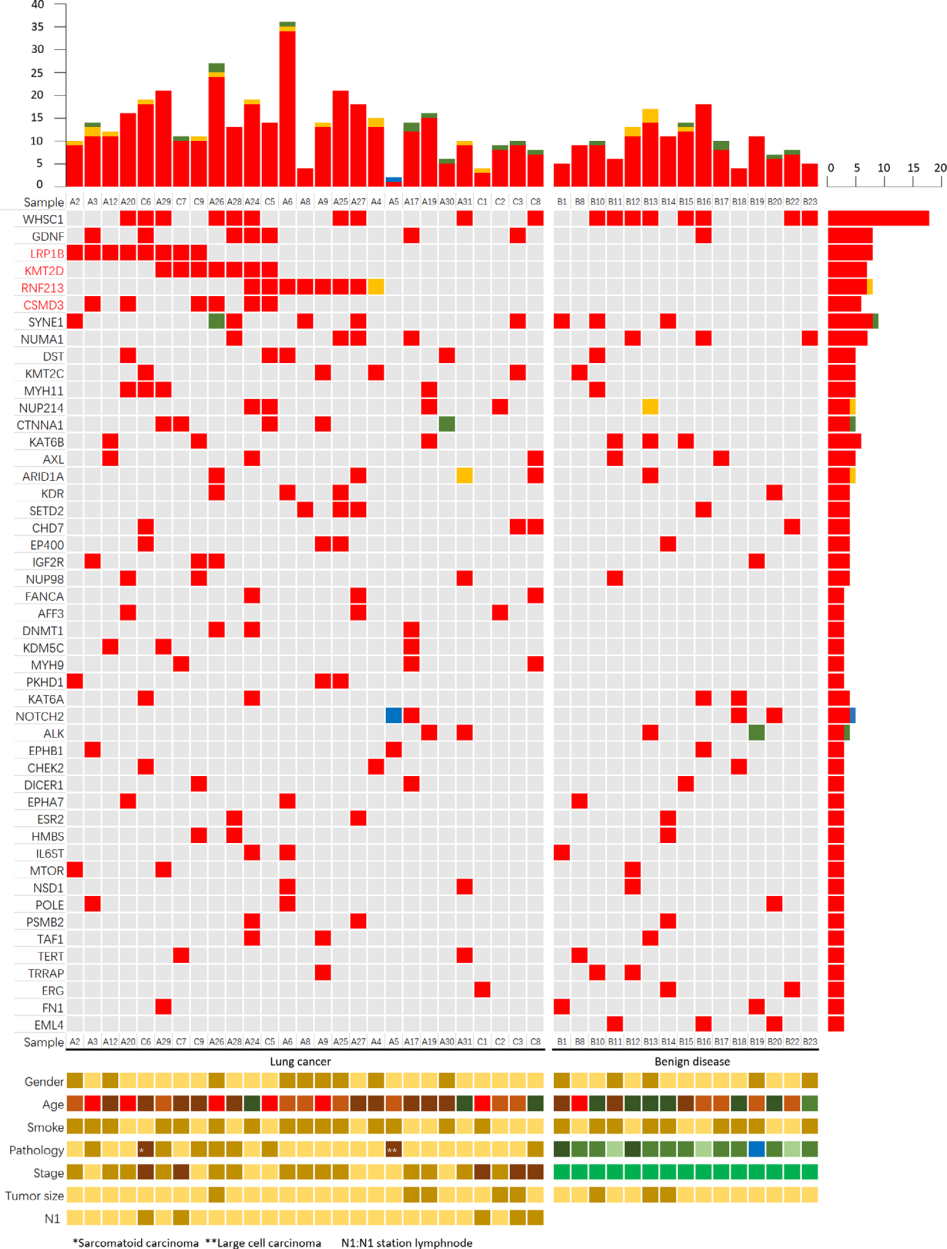
Heat map of somatic SNVs in the lung cancer and benign disease groups of the training set. The most frequently mutated gene was WHSC1. There were 10 and eight samples mutated, respectively, in the two groups (*P* > 0.05). RNF213, KMT2D, CSMD3 and LRP1B were more frequently mutated in lung cancer than benign disease (*P* < 0.05). The genes mutated in fewer than three patients are not listed in the heat map (all data are shown in Supporting Information). Mutation type: (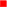) Missense mutation, (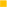) Nonsense mutation, (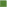) Splice site, (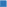) Del. Gender: (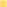) Male, (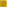) Female. Age: (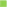) 20–30, (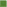) 31–40, (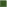) 41–50, (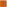) 51–60, (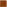) 61–70, (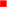) 70–. Smoke: (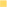) Smoke, (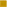) No smoke. Pathology: (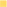) Adenocarcinoma, (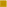) Squamous‐cell carcinoma, (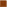) Other NSCLC, (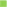) Tuberculosis, (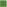) Inflammation, (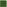) Hamartoma, (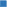) Sclerosing angioma. Stage: (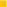) IA stage, (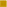) IB stage, (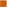) IIA stage, (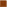) IIB stage, (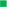) Benign. Tumor size: (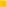) Tumor ≤3 cm, (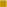) 3 cm <Tumor ≤4 cm. N1: (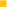) −; (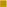) +

The 42 sequenced samples were divided into lung cancer and benign disease groups according to postoperative pathology. There were 27 samples in the lung cancer group and 15 samples in the benign disease control group. Somatic mutations were detected in both groups. In total, 246 gene mutations were detected in the training set (Fig 3). The number of missense mutations in the *LRP1B*, *KMT2D*, *RNF213* and *CSMD3* genes was eight (8/27, 29.6%), seven (7/27, 25.9%), seven (7/27, 25.9%) and six (6/27, 22.2%) in the lung cancer group, respectively, and none of the genes were mutated in the benign disease control group.

*LRP1B*, *KMT2D*, *RNF213* and *CSMD3* gene mutations were significantly more common in lung cancer than in benign disease (*P* < 0.05). The *WHSC1* gene was mutated in 10 samples of lung cancer and eight samples of benign disease. The *GDNF* gene was mutated in seven lung cancer samples and one benign disease sample. There were fewer than five mutations of other genes detected in either of the two groups. There were no significant differences in these gene mutation rates between the two groups (*P* > 0.05).

### Immunohistochemical results

**Figure 4:**
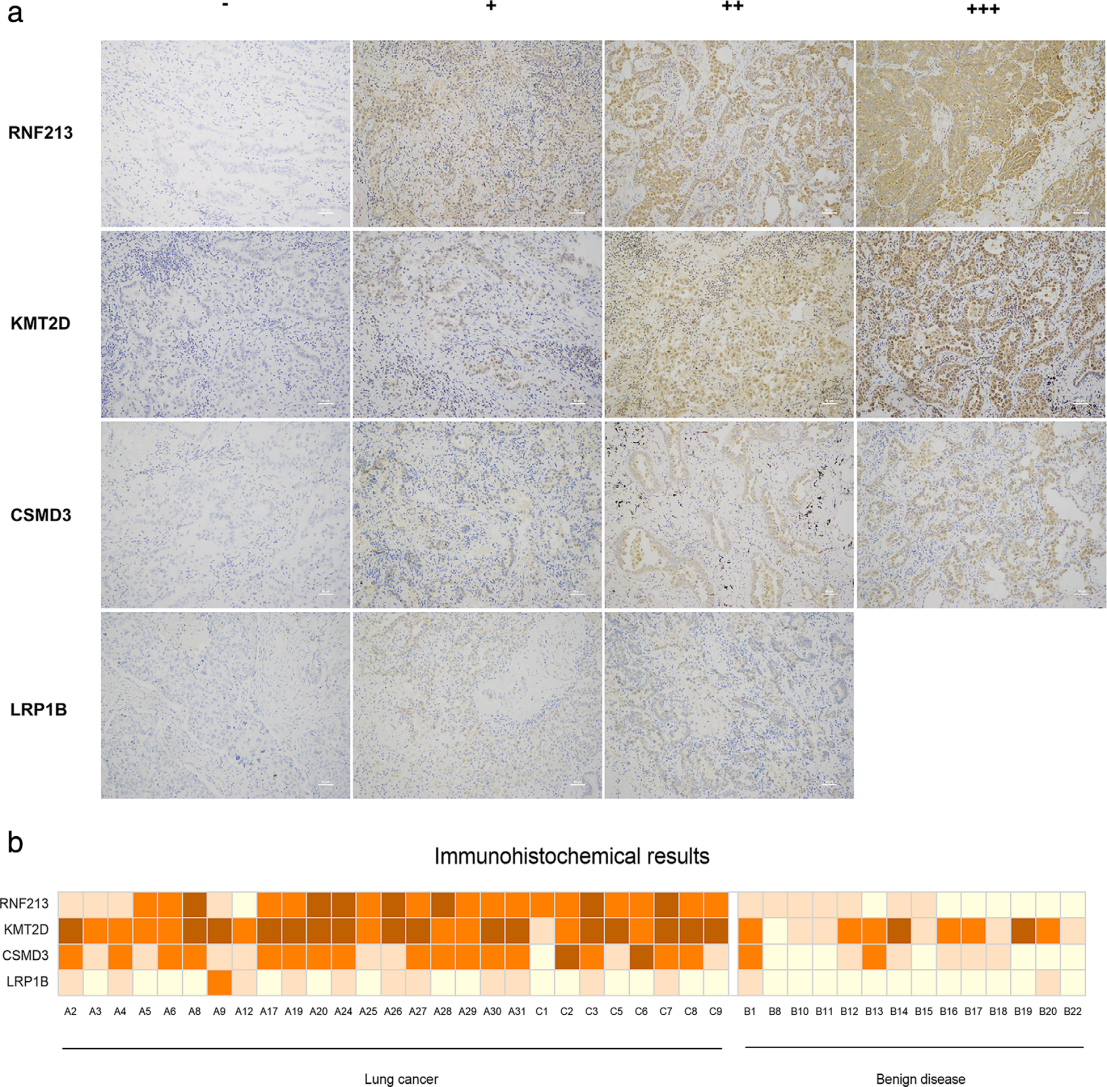
The results of immunohistochemistry. (**a**) Representative illustrations of the expression of RNF213, KMT2D, CSMD3 and LRP1B in lung cancer and benign disease. “−” and “+” represent low expression of RNF213, KMT2D, CSMD3 and LRP1B (IHC, ×200). “++” and “+++” represent high expression of these four genes (IHC, ×200). (**b**) The summary of RNF213, KMT2D, CSMD3 and LRP1B expression in 27 lung cancer and 14 benign disease tissues. (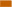) +++, (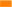) ++, (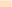) +, (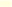) −

In the training set, the *RNF213*, *LRP1B*, *KMT2D* and *CSMD3* genes had statistically significant differences in the sequenced data in lung cancer compared to benign disease. Immunohistochemistry (IHC) was performed on specimens of 27 lung cancer and 14 benign disease FFPE tissues to detect the expression of *RNF213*, *LRP1B*, *KMT2D* and *CSMD3*. IHC was not carried out in one sample (B23) because the tissue was too small. After staining, photography and evaluation, we chose representative illustrations of the expression of *RNF213*, *KMT2D*, *CSMD3* and *LRP1B* (Fig 4). High and low expression levels of the four genes are summarized in Fig 4. High expression of *RNF213*, *KMT2D* and *CSMD3* was observed in lung cancer tissues, and low expression of these genes was observed in benign disease tissues. There were significant differences in their levels between the two groups (*P* < 0.05), especially *RNF213* (*P* < 0.005). Low expression of LRP1B was observed in 26 lung cancer tissues and in 14 benign disease tissues. One of the lung cancer samples was highly expressed. This result was not significant (*P* > 0.05).

### Validation set results

**Figure 5:**
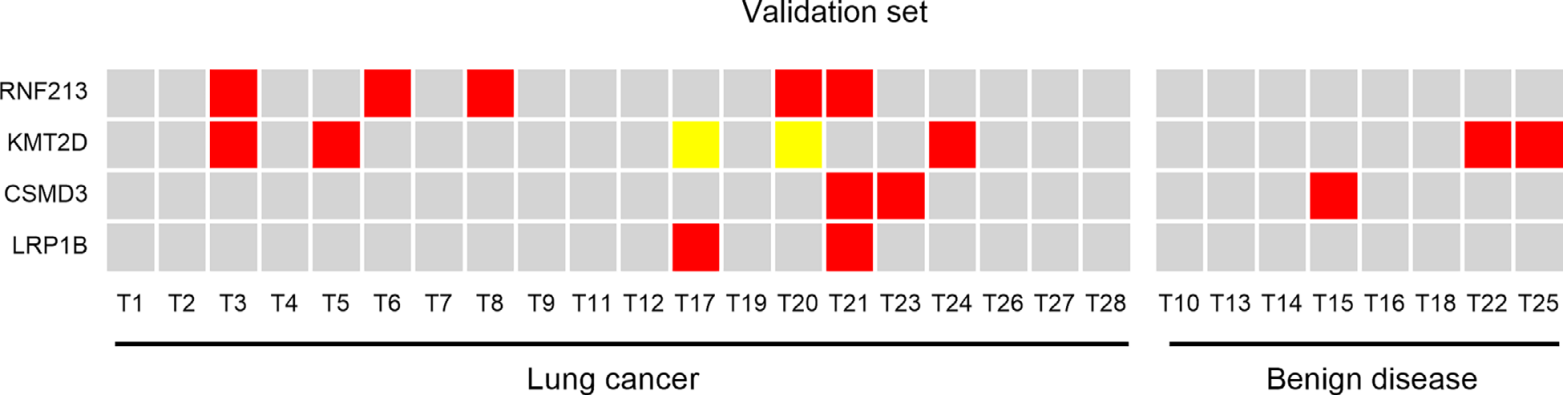
Mutations in the validation set. RNF213 gene mutations were detected in five lung cancer samples and none in the benign control group. This is consistent with the data in the training set. (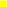) Nonsense mutation, (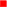) Missense mutation, (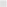) No mutation

There were 28 pulmonary nodule patients enrolled in the validation set without a definite diagnosis through chest CT. The largest diameter of the lesion was less than 3 cm on CT imaging in each case. Malignant or benign nodules could not be confirmed by CT imaging or biomarkers. Blood samples were submitted to targeted sequencing using the same method. The number of the test genes *RNF213*, *KMT2D*, *CSMD3* and *LRP1B* detected in lung cancer samples was five, five, three and two, respectively. A total of 20 samples were confirmed to be lung cancer and eight samples were benign nodules on postoperative pathology. The R*NF213* gene was mutated in 25% of lung cancer patients. *KMT2D*, *CSMD3* and *LRP1B* were mutated in 15%, 10% and 10% of lung cancer patients, but *KMT2D* and *CSMD3* were mutated in 25% and 12.5% of benign diseases (Fig 5).

### Analysis of GGO and all samples

We analyzed all sequenced data of the 70 samples (42 training set samples and 28 verification set samples). *RNF213* gene mutated in 12 lung cancer (25.5%) and 0 benign disease samples. *RNF213* gene mutated more frequently in lung cancer than benign diseases (*P* = 0.006, two‐tailed). In addition, there were 55 patients diagnosed with GGO on chest CT, including 36 with early‐stage lung cancer and 19 with benign disease. We detected *RNF213* gene mutations in 10 (10/36, 27.8%) lung cancer samples and no samples in the benign disease group (*P* < 0.05). All of these somatic mutations were missense mutations. The specificity of the *RNF213* gene mutation was 100% in the diagnosis of GGO, and its sensitivity was 27.8%.

## Discussion

Early diagnosis and treatment are effective means to improve the survival rate of patients with early stage lung cancer. Small lung lesions can be found on chest CT, which is the most common and valid examination modality in the diagnosis or screening of lung cancer.^25^, ^26^ Recently, early detection or screening with low‐dose computed tomography (LDCT) by the National Lung Screening Trial (NLST) and other studies has been shown to improve survival and reduce lung cancer‐specific mortality.^27^, ^28^

Some lesions are easy to diagnose as lung cancer on CT, and some lesions are difficult to identify as lung cancer or benign disease, especially when the pulmonary nodule is small or imaging features are not typical.^29^ For example, a ground‐glass opacity (GGO) or ground‐glass nodule (GGN) is usually adenocarcinoma in situ (AIS), minimally invasive adenocarcinoma (MIA) or atypical adenomatous hyperplasia (AAH).^30^ The higher false positive and false negative rate using traditional biomarkers such as carcinoembryonic antigen (CEA), neuron‐specific enolase (NSE) and cytokeratin 19 (CYFRA‐211) make the diagnosis more difficult.^31^, ^32^ These biomarkers are useless in the early diagnosis of lung cancer. Aspiration biopsy may be needed to confirm that the nodule is a malignancy or benign disease. However, bleeding, pneumothorax, pain and possible diversion restrict its use in early diagnosis.

An ideal diagnostic method should be simple, convenient, safe, and efficient. Using liquid biopsies to detect circulating biomarkers such as circulating tumor cells (CTCs), circulating tumor DNA (ctDNA) and exosomes may offer a relatively simple method to analyze early‐stage tumors.^33^ Detecting ctDNA in peripheral blood is used more commonly, while CTCs are less commonly detected in early‐stage tumors.

Circulating cell‐free DNA, a fragment of DNA that is released through cell apoptosis, widely exists in extracellular fluid, such as blood, cerebrospinal fluid, urine and saliva.^4^, ^5^ The cfDNA of healthy people comes mainly from metabolism and cell apoptosis, including bone marrow cells, lymphocytes, and normal tissue cells.^34^ For patients with tumors, the cfDNA fragments of tumor cells, known as circulating tumor DNA (ctDNA), are also released into peripheral blood through apoptosis and necrosis of tumor cells.^6^, ^7^ Plasma ctDNA, which is a fragment of approximately 150–200 bp^35^ containing genetic information about the tumor, is of great significance for the diagnosis, treatment and monitoring of the disease.

Circulating tumor DNA is used to monitor the therapeutic effects and make prognostic predictions in the treatment of malignancies because of the ctDNA levels in advanced‐stage tumors.^36^The level of the detected ctDNA increase correlates with malignant progression.^13^, ^37^ The low levels of ctDNA in early‐stage tumors make detection difficult. Early diagnosis can provide tremendous benefits for the treatment of patients with malignant tumors.^13^, ^38^ With the development of sequencing technology, low levels of ctDNA could be detected in blood more easily and accurately. More and more studies have applied this technology to investigate early‐stage tumors.

In the present study, we investigated ctDNA in early‐stage lung cancer and comparable benign disease diagnosed by chest CT. First, cfDNA was detected in all benign lung disease samples, as reported previously in other solid tumors.^39^, ^40^, ^41^ However, these studies did not consider the stage of malignancy compared to benign disease. The level of cfDNA in malignancy is related to the tumor burden, as measured by such indices as tumor size, T stage and TNM stage.^42^ Our data indicated that the level of cfDNA in early‐stage lung cancer was not significantly different from that of benign disease (0.53 ± 0.13 ng/μL and 0.54 ± 0.07 ng/μL, respectively; *P* > 0.05). This result may be related to the low tumor burden in early‐stage lung cancer, indicating that early‐stage lung cancers release low levels of cfDNA into the blood stream, similarly to benign lung disease. Cell apoptosis and necrosis from benign tumors or diseases also cause cfDNA to increase.

Elevated cfDNA concentrations alone do not fully distinguish between lung cancer and benign disease. In our study, targeted NGS was implemented to detect ctDNA in these DNA samples. The panel used for NGS covered all known mutated genes in malignant tumors to investigate mutations in early‐stage lung cancer. We found that the number of mutations was not related to sex, age, smoking history, tumor size, stage or pathology in the two groups. Some genes were mutated more frequently in lung cancer and others in benign disease. In the training set, *RNF213*, *KMT2D*, *CSMD3* and *LRP1B* were mutated more frequently in early‐stage lung cancer than in benign disease. A total of 25.9% of lung cancer patients showed *RNF213* gene mutations, and no lung cancer was observed in benign disease patients. The *RNF213* gene had a high specificity in distinguishing lung cancer from benign disease.

To clarify the protein expression of these four genes in tissues, we conducted immunohistochemistry of lung cancer tissues. *RNF213*, *KMT2D* and *CSMD3* genes showed higher expression than in benign disease samples, especially *RNF213*. This differential expression between the two groups may be due to the changes in amino acids caused by the genetic changes.

Finally, a verification experiment was carried out to study the assisted diagnostic value of these four genes. NGS was also used in the validation set as in the training set. The *RNF213* gene was mutated in 25% of lung cancer patients and was not mutated in benign diseases, but *KMT2D*, *CSMD3* and *LRP1B* were mutated less in both groups. The same high specificity of the *RNF213* gene mutation was shown in the validation set.

Considering the difference of *RNF213* gene was not statistically significant in the validation set probably due to the small number of samples, we analyzed all 70 samples of the study to confirm those which were statistically significant. There were 47 lung cancer and 23 benign disease in all samples. *RNF213* gene mutated in 12 lung cancer (25.5%) and 0 benign disease samples. In all 70 samples of our study, *RNF213* gene mutated more frequently in lung cancer than benign diseases (*P* = 0.006, two ‐tailed). Theoretically, increasing the sample size of the validation set results in statistical difference. In other words, all patients with *RNF213* gene wild‐type in peripheral blood had benign disease in our study and about 25% patients with *RNF213* gene mutant type were lung cancer.

In addition, we investigated *RNF213* in all GGO patients in the study. The results showed that the *RNF213* gene was mutated in 27.8% of lung cancer samples and in none of the benign disease samples (*P* < 0.05), with a similarly high specificity. Our sample number was low, and a greater number of randomized controlled samples are needed to further confirm these results.

The *RNF213* gene, encoding ring finger protein 213, encodes a protein containing a RING finger domain.^43^ It is mutated in some malignant tumors, such as ovarian cancer, gastric cancer and liver cancer,^44^, ^45^, ^46^ yet there have been few studies on *RNF213* gene mutations in malignant tumors. *RNF213* has been reported to be a tumor suppressor in malignancy.^47^ We first found an *RNF213* gene mutation in ctDNA of early‐stage lung cancer, and this mutation was found at a significantly different rate than in benign lung disease. This missense mutation of *RNF213* changed the amino acids, thus affecting the protein function. This gene mutation may result in a loss of tumor suppressor function and promote tumor development and progression in lung cancer. The mechanisms need to be determined in the future.

In conclusion, the concentration of cfDNA is not a good biomarker for the assisted diagnosis of early‐stage lung cancer and benign lung diseases. *RNF213* gene mutation in ctDNA may be used for the molecular diagnosis of malignant and benign lung nodules. It has a high specificity of 100% in the assisted diagnosis of lung cancer compared to benign lung disease in pulmonary nodules. The results of our study may be useful for the assisted early diagnosis of lung cancer. The effect of KMT2D, CSMD3 and LRP1B should be further confirmed in more samples.

The shortcoming of this study is the small sample size, the reason for which is lack of research funding. In the training set, five patients which were considered clinical stage I lung cancer due to chest CT were upgraded to stage IIB because the N1 station lymph node was positive. A larger scale randomized controlled trial is needed to verify this finding in the future. In addition, the underlying mechanisms of the *RNF213* gene in the development of lung cancer requires further study.

## Disclosure

The authors declare no competing interests.

## Supporting information


**Figure S1** Heat map of somatic SNVs in the lung cancer and benign disease groups of the training set. Somatic mutations were detected by targeted NGS in lung cancer and benign disease.

**Table S2** Somatic mutations of training set.

**Table S3** Somatic mutations of validation set.

**Table S4** Mutation sites of significant genes in lung cancer.
