# Supplementary figures and images for: RNF213 gene mutation in circulating tumor DNA detected by targeted next‐generation sequencing in the assisted discrimination of early‐stage lung cancer from pulmonary nodules
Source: Thorac Cancer. 2020 Nov 16;12(2):181–93. doi: 10.1111/1759-7714.13741 (PMC7812078; doi:10.1111/1759-7714.13741)

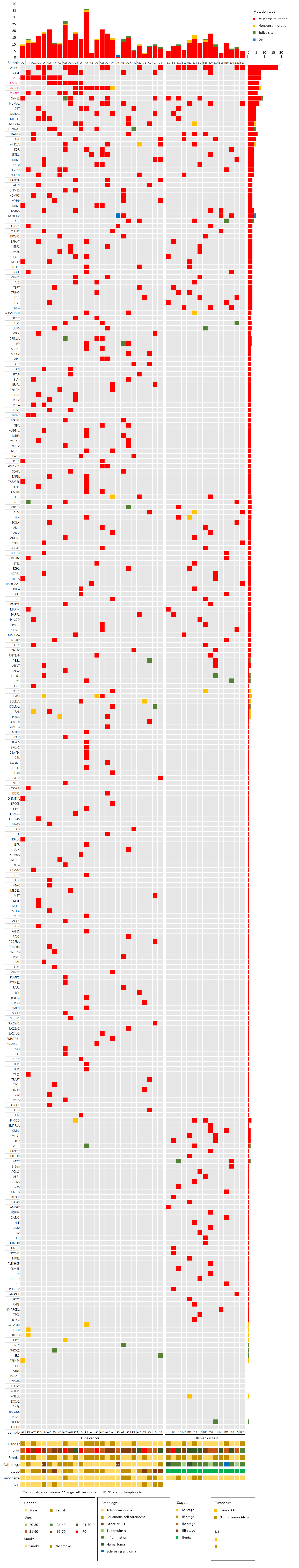

Supplement: Supplementary file 1 — Figure S1 Heat map of somatic SNVs in the lung cancer and benign disease groups of the training set. Somatic mutations were detected by targeted NGS in lung cancer and benign disease. [file TCA-12-181-s001.tif]
