# Supplementary material for: RNF213 gene mutation in circulating tumor DNA detected by targeted next‐generation sequencing in the assisted discrimination of early‐stage lung cancer from pulmonary nodules
Source: Thorac Cancer. 2020 Nov 16;12(2):181–93. doi: 10.1111/1759-7714.13741 (PMC7812078; doi:10.1111/1759-7714.13741)
Supplement: Supplementary file 4 — Table S4 Mutation sites of significant genes in lung cancer. [file TCA-12-181-s004.docx]

Table 3: Mutation sites of significant genes in lung cancer

| Gene | Chromosome | Exon | cDNA | Amino acid | Type | Num. |
| --- | --- | --- | --- | --- | --- | --- |
| RNF213 | 17 | 26 | c.G5960A | p.G1987E | Missense | 1 |
|  | 17 | 26 | c.C5171T | p.A1724V | Missense | 2 |
|  | 17 | 26 | c.C5155T | p.Q1719X | Nonsense | 1 |
|  | 17 | 59 | c.C14226A | p.S4742R | Missense | 1 |
|  | 17 | 29 | c.A8252T | p.N2751I | Missense | 1 |
|  | 17 | 17 | c.A3101T | p.K1034M | Missense | 1 |
|  | 17 | 24 | c.G4615A | p.A1539T | Missense | 1 |
| KMT2D | 12 | 34 | c.C8972T | p.P2991L | Missense | 1 |
|  | 12 | 34 | c.C8495T | p.A2832V | Missense | 2 |
|  | 12 | 16 | c.C4474T | p.Q1492X | Nonsense | 1 |
|  | 12 | 4 | c.G487A | p.A163T | Missense | 1 |
|  | 12 | 41 | c.G13711A | p.A4571T | Missense | 1 |
|  | 12 | 11 | c.C3647T | p.A1216V | Missense | 1 |
|  | 12 | 38 | c.G10607A | p.R3536H | Missense | 1 |
| CSMD3 | 8 | 54 | c.C8300T | p.P2767L | Missense | 1 |
|  | 8 | 20 | c.A3058T | p.T1020S | Missense | 3 |
|  | 8 | 14 | c.C2050T | p.L684F | Missense | 1 |
|  | 8 | 10 | c.G1397C | p.S466T | Missense | 1 |
|  | 8 | 59 | c.G9323T | p.G3108V | Missense | 1 |
| LRP1B | 2 | 51 | c.G8185A | p.A2729T | Missense | 1 |
|  | 2 | 83 | c.A12658T | p.T4220S | Missense | 1 |
|  | 2 | 2 | c.A125T | p.H42L | Missense | 3 |
|  | 2 | 16 | c.G2555A | p.C852Y | Missense | 1 |
|  | 2 | 89 | c.A13511T | p.H4504L | Missense | 1 |
|  | 2 | 8 | c.A1141T | p.N381Y | Missense | 1 |
